# An efficient expression tag library based on self-assembling amphipathic peptides

**DOI:** 10.1186/s12934-019-1142-9

**Published:** 2019-05-27

**Authors:** Weixin Zhao, Song Liu, Guocheng Du, Jingwen Zhou

**Affiliations:** 1 National Engineering Laboratory for Cereal Fermentation Technology, Jiangnan University, Wuxi, 214122 China; 2 School of Biotechnology, Jiangnan University, Wuxi, 214122 China; 3 The Key Laboratory of Carbohydrate Chemistry and Biotechnology, Ministry of Education, Jiangnan University, Wuxi, 214122 China; 4 Jiangsu Provisional Research Center for Bioactive Product Processing Technology, Jiangnan University, Wuxi, 214122 China

**Keywords:** Self-assembling amphipathic peptides, Expression tags, Hydrophobicity, Positive charge, High-throughput screening

## Abstract

**Background:**

Self-assembling amphipathic peptides (SAPs) may improve protein production or induce the formation of inclusion bodies by fusing them to the N-terminus of proteins. However, they do not function uniformly well with all target enzymes and systematic research on how the composition of SAPs influence the production of fusion protein is still limited.

**Results:**

To improve the efficiency of SAPs, we studied factors that might be involved in SAP-mediated protein production using S1 (AEAEAKAK)_2_ as the original SAP and green fluorescent protein (GFP) as the reporter. The results indicate that hydrophobicity and net charges of SAPs play a key role in protein expression. As hydrophobicity regulation tend to cause the formation of insoluble inclusion bodies of protein, an expression tag library composed of SAPs, which varied in net charge (from + 1 to + 20), was constructed based on the random amplification of S1*nv*1 (ANANARAR)_10_. The efficiency of the library was validated by polygalacturonate lyase (PGL), lipoxygenase (LOX), l-asparaginase (ASN) and transglutaminase (MTG). To accelerate preliminary screening, each enzyme was fused at the C-terminus with GFP. Among the four enzyme fusions, the SAPs with + 2 – + 6 net charges were optimal for protein expression. Finally, application of the library improved the expression of PGL, LOX, ASN, and MTG by 8.3, 3.5, 2.64, and 3.68-fold relative to that of the corresponding wild-type enzyme, respectively.

**Conclusions:**

This is the first report to study key factors of SAPs as an expression tag to enhance recombinant enzyme production. The SAP library could be used as a novel plug-and-play protein-engineering method to screen for enzymes or proteins with enhanced production.

**Electronic supplementary material:**

The online version of this article (10.1186/s12934-019-1142-9) contains supplementary material, which is available to authorized users.

## Background

*Escherichia coli* is preferred for heterologous protein production because of its short growth period, simple transformation process, uncomplicated nutritional and sterility requirements, and extensive research foundation [1]. To improve the protein production in this expression system, efforts have been directed toward promoter screening [2], N-terminal codon optimization [3], fusing with an expression tag at the terminus [4], and culture condition optimization [5].

Due to the high efficiency of protein production, a series of tags have been developed, including glutathione S-transferase (GST) [6], maltose binding protein (MBP) [7], small ubiquitin modifier (SUMO) [8], and N-utilization substance (NusA) [9]. These proteins or tags could regulate the process of protein transcription and translation [10] or help to fold the protein properly [4], thus leading to enhanced expression of the target protein. However, a specific fusion tag does not work efficiently in all cases [11], and the biological activity of functional proteins could even be inhibited by the fused tags [12]. Thus, it is desirable to improve the universality of expression tags without sacrificing the biological properties of the target proteins.

Self-assembling amphipathic peptides (SAPs) are short peptides constituted by alternating hydrophobic and hydrophilic residues [13]. We previously showed that S1 (AEAEAKAK)_2_, a SAP originating from the Zuotin protein sequence, improved the production and thermal stability of the LOX protein when fused to its N-terminus in *E. coli* [14]. The positive effects of SAPs on enzyme activity or stability were also observed in the SAP fusions of amylase [15] and nitrile hydratase [16]. We then generated an S1 variant (AEAEAHAH)_2_ with the ability to benefit the production, thermal stability, and purification yield of recombinant proteins in *E. coli* [17]. These findings of positive effects on both protein production and properties suggested that the desired expression tags could be derived from SAPs.

Although there is a certain degree of universality for enhancing protein production, fusion with SAPs could not improve the production of all proteins to an acceptable extent in *E. coli* [17]. The mechanism by which the production enhancement function of SAPs can be maximized remains unexplored. It was reported that a SAP (LELELKLK)_2_ with high hydrophobicity induced in vivo assembly of active protein aggregates after fusing to the terminus of proteins in *E. coli* [18]. These findings implied that the hydrophobicity or charges of the SAPs play an important role in the production of protein fusions. In addition, as the bridge of the fusion protein, linker regions are also important for the construction of bioactive fusion proteins, with the length and rigidity of linker peptides directly affecting the orientation of the linked proteins [19]. Based on these reports, SAP fusion may be optimized for enhancing the production of a specific protein by adjusting SAP and linker compositions.

Here we explored the factors that could increase the efficiency of SAPs as an expression fusion tag in *E. coli*. First, we studied the key factors (the composition of SAPs and linker peptides) that might be involved in SAP fusion production using GFP as a reporter. Then, an expression tag library composed of SAPs which varied in net charge was constructed based on the random amplification of S1*nv*1 (ANANARAR)_10_, an S1 variant. Finally, the efficiency of the library was validated using four different enzymes.

## Materials and methods

### Strains and plasmids

**Fig. 1:**
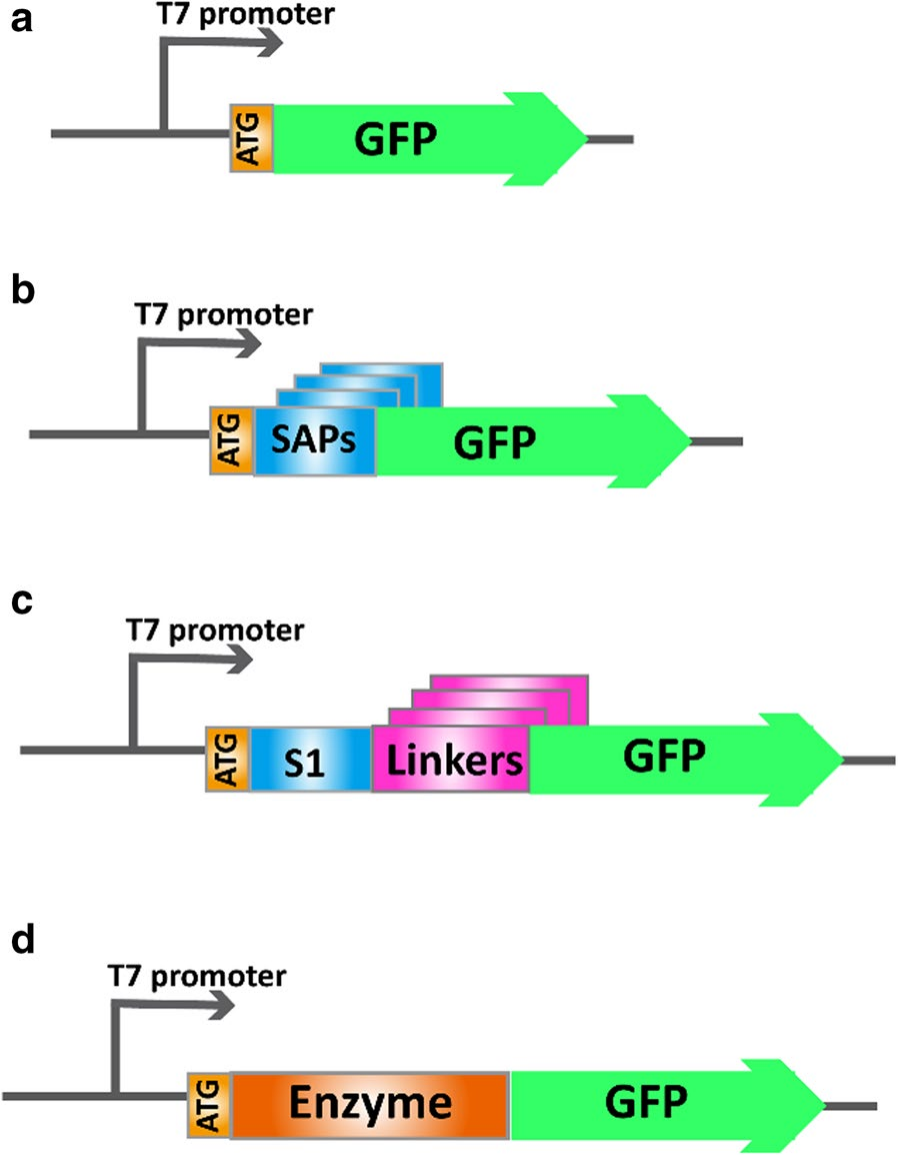
Schemes for the construction of expression plasmids. **a** The expression plasmid for wild-type GFP. **b** The expression plasmids for GFP fused with different SAPs. **c** The expression plasmids for GFP fused with S1 via different linker peptides. **d** The expression plasmid for enzyme fused with GFP

*Escherichia coli* JM109 and *E. coli* BL21 (DE3) cells (Novagen, Madison, WI, USA) were used for gene cloning and protein expression, respectively. Plasmid pET-22b( + )/*gfp* (Fig. 1a) encoding wild-type GFP (*gfp*) from *Aequorea victoria* [20] was constructed as described previously [17].

### Plasmid construction

The gene fragments of S1 and its derivatives, which varied in hydrophobic residues (Table 1, S1*hv*1–S1*hv*6), hydrophilic residues (Table 1, S1*cv*1–S1*cv*5), and net charge (Table 1, S1*nv*1 and S1*nv*2), were chemically synthesized and cloned into the *Nde*I and *Nco*I sites of pET-22b(+)/*gfp* by Sangon Biotech (Shanghai, China), yielding the plasmids expressing GFP fusions with different SAPs at the N-terminus (Fig. 1b). To avoid the effect of codons on protein expression, all genes encoding SAPs were synthesized according to the codon usage preferred by *E. coli* [21].

**Table 1:** The amino acid sequence of SAPs designed in this study

Schemes	SAPs	Amino acid sequence	GRAVY^a^	Net charge
	S1	(AEAEAKAK)_2_	− 0.95	0
Hydrophobic residues	S1*hv*1	(IEIEIKIK)_2_	0.4	0
	S1*hv*2	(LELELKLK)_2_	0.05	0
	S1*hv*3	(VEVEVKVK)_2_	0.25	0
	S1*hv*4	(FEFEFKFK)_2_	− 0.45	0
	S1*hv*5	(GEGEGKGK)_2_	− 2.05	0
	S1*hv*6	(PEPEPKPK)_2_	− 2.65	0
Hydrophilic residues	S1*cv*1	(ADADAHAH)_2_	− 0.78	0
	S1*cv*2	(AEAEAHAH)_2_	− 0.78	0
	S1*cv*3	(ADADAKAK)_2_	− 0.95	0
	S1*cv*4	(AEAEARAR)_2_	− 1.1	0
	S1*cv*5	(ADADARAR)_2_	− 1.1	0
Length	S1*lv*1	AEAEAKAK	− 0.95	0
	S1*lv*2	(AEAEAKAK)_3_	− 0.95	0
	S1*lv*3	(AEAEAKAK)_4_	− 0.95	0
	S1*lv*4	(AEAEAKAK)_5_	− 0.95	0
	S1*lv*5	(AEAEAKAK)_6_	− 0.95	0
	S1*lv*6	(AEAEAKAK)_7_	− 0.95	0
	S1*lv*7	(AEAEAKAK)_8_	− 0.95	0
	S1*lv*8	(AEAEAKAK)_9_	− 0.95	0
	S1*lv*9	(AEAEAKAK)_10_	− 0.95	0
Net charge	S1*nv*1	(ANANARAR)_10_	− 1.1	+ 20
	S1*nv*2	(ANANADAD)_10_	− 1.1	− 20
	S1*nv*3	(ANANADAD)_8_	− 1.1	− 16
	S1*nv*4	(ANANADAD)_7_	− 1.1	− 14
	S1*nv*5	(ANANADAD)_6_	− 1.1	− 12
	S1*nv*6	(ANANADAD)_4_	− 1.1	− 8
	S1*nv*7	(ANANADAD)_3_	− 1.1	− 6
	S1*nv*8	(ANANADAD)_2_	− 1.1	− 4
	S1*nv*9	ANANADAD	− 1.1	− 2
	S1*nv*10	(ANANARAR)_2_	− 1.1	+ 4
	S1*nv*11	(ANANARAR)_3_	− 1.1	+ 6
	S1*nv*12	(ANANARAR)_4_	− 1.1	+ 8
	S1*nv*13	(ANANARAR)_5_	− 1.1	+ 10
	S1*nv*14	(ANANARAR)_6_	− 1.1	+ 12
	S1*nv*15	(ANANARAR)_8_	− 1.1	+ 16
	S1*nv*16	(ANANARAR)_9_	− 1.1	+ 18
	S1*nv*17	ANANARARANANAR	− 1.064	+ 3

^a^The total average of hydrophobicity (GRAVY, https://web.expasy.org/) was used to indicate the hydrophobicity of SAPs

DNA manipulations in our study were based on standard protocols and the related primer pairs are shown in Additional file 1: Table S1. The plasmids expressing the GFP fusions containing different S1 units (Table 1, S1*lv*1–S1*lv*9) (Fig. 1b) were constructed by whole plasmid polymerase chain reaction (PCR) as shown in Additional file 1: Figure S1. The plasmid encoding GFP fused with S1 (Table 1, Additional file 1: Table S1) was used as the template and S1*lv*1-F/*S1*-R were the primer pairs. PCR was conducted using the followed amplification program: an initial denaturation of 3 min at 98 °C, followed by 34 cycles of 10 s at 98 °C, 10 s at 55 °C, and 6 min at 72 °C. The length of SAP may be altered by changing the PCR annealing temperature, yielding several GFP fusions containing different number of S1 units. Ten fusions with SAPs ranging from a half to five S1 units (Table 1, S1*lv*1–S1*lv*9) were selected.

Plasmids expressing GFP fused with S1*nv*1 were used as the templates to construct the fusions containing SAPs carrying different positive net charges (Table 1, S1*nv*10–S1*nv*17) using the primer pairs *S1nv*1-F/*S1*-R. Plasmids expressing GFP fused with S1*nv*2 were used as templates to construct fusions containing SAPs carrying different negative net charges (Table 1, S1*nv*3–S1*nv*9) using the primer pairs *S1nv*2-F/*S1*-R. The mutation of the net charge variation (Fig. 1b) were introduced through whole plasmid PCR using the corresponding templates and primers, and PCR was conducted using the followed amplification program: initial denaturation of 3 min at 98 °C, followed by 34 cycles of 10 s at 98 °C, 10 s at 55 °C, and 6 min at 72 °C. To remove the primary template, *Dpn*I was used to digest the PCR products, followed by purification and ligation by T4 ligase (Shanghai, China) to obtain circular plasmids. Then the circular plasmids were transformed into *E. coli* BL21 (DE3) for GFP fusion expression.

The gene fragments of linker peptides (Table 2) were separately synthesized and inserted into the *Nco*I site of the plasmids of GFP fused with S1 by Sangon Biotech (Shanghai, China), yielding the plasmids expressing GFP fused with S1 via different linkers (Fig. 1c). To avoid the effect of codons on the protein expression, all of the genes encoding linkers were synthesized according to the codon usage preferred by *E. coli* [21].

**Table 2:** Amino acid sequences of the linker peptides designed in this study

Linkers	Linker sequences
1	EAAAK
2	EAAAKEAAAK
3	EAAAKEAAAKEAAAK
4	EAAAKEAAAKEAAAKEAAAK
5	EAAAKEAAAKEAAAKEAAAKEAAAK
6	EAAAKEAAAKEAAAKEAAAKGGGGS
7	EAAAKEAAAKEAAAKGGGGSGGGGS
8	EAAAKEAAAKGGGGSGGGGSGGGGS
9	EAAAKGGGGSGGGGSGGGGSGGGGS
10	EAAAKGGGGSGGGGSGGGGSEAAAK
11	EAAAKGGGGSGGGGSEAAAKGGGGS
12	EAAAKEAAAKGGGGSGGGGSEAAAK
13	EAAAKGGGGSGGGGSEAAAKEAAAK
14	EAAAKGGGGSEAAAKEAAAKEAAAK
15	EAAAKEAAAKGGGGSEAAAKEAAAK
16	EAAAKEAAAKEAAAKGGGGSEAAAK
17	EAAAKGGGGSEAAAKGGGGSEAAAK
18	EAAAKEAAAKGGGGSEAAAKGGGGS
19	EAAAKGGGGSEAAAKGGGGSGGGGS
20	EAAAKEAAAKGGGGSGGGGSEAAAK
21	GGGGS
22	GGGGSGGGGS
23	GGGGSGGGGSGGGGS
24	GGGGSGGGGSGGGGSGGGGS
25	GGGGSGGGGSGGGGSGGGGSGGGGS
26	GGGGSGGGGSGGGGSGGGGSEAAAK
27	GGGGSGGGGSGGGGSEAAAKEAAAK
28	GGGGSGGGGSEAAAKEAAAKEAAAK
29	GGGGSEAAAKEAAAKEAAAKEAAAK
30	GGGGSEAAAKEAAAKEAAAKGGGGS
31	GGGGSEAAAKEAAAKGGGGSEAAAK
32	GGGGSGGGGSEAAAKEAAAKGGGGS
33	GGGGSEAAAKEAAAKGGGGSGGGGS
34	GGGGSEAAAKGGGGSGGGGSGGGGS
35	GGGGSGGGGSEAAAKGGGGSGGGGS
36	GGGGSGGGGSGGGGSEAAAKGGGGS
37	GGGGSEAAAKGGGGSEAAAKGGGGS
38	GGGGSGGGGSEAAAKGGGGSEAAAK
39	GGGGSEAAAKGGGGSEAAAKEAAAK
40	GGGGSGGGGSEAAAKEAAAKGGGGS

The rigid unit was (EAAAK) while the flexible unit was (GGGGS)

### Library construction for screening fusions with enhanced production

The PGL gene from the genome of *Bacillussubtilis* WSHB04-02 (CCTCCM 204082), LOX from *Pseudomonas aeruginosa* BBE (CCTCC M2011185), ASN from *E. coli* (*E. coli* strain BL21, TaKaRa), and MTG from *Streptomycesmobaraense* (CICC 11018) were amplified using the corresponding primer pairs *pgl*-F/*pgl*-R, *lox*-F/*lox*-R, *asn*-F/*asn*-R, and *mtg*-F/*mtg*-R, respectively.

**Fig. 2:**
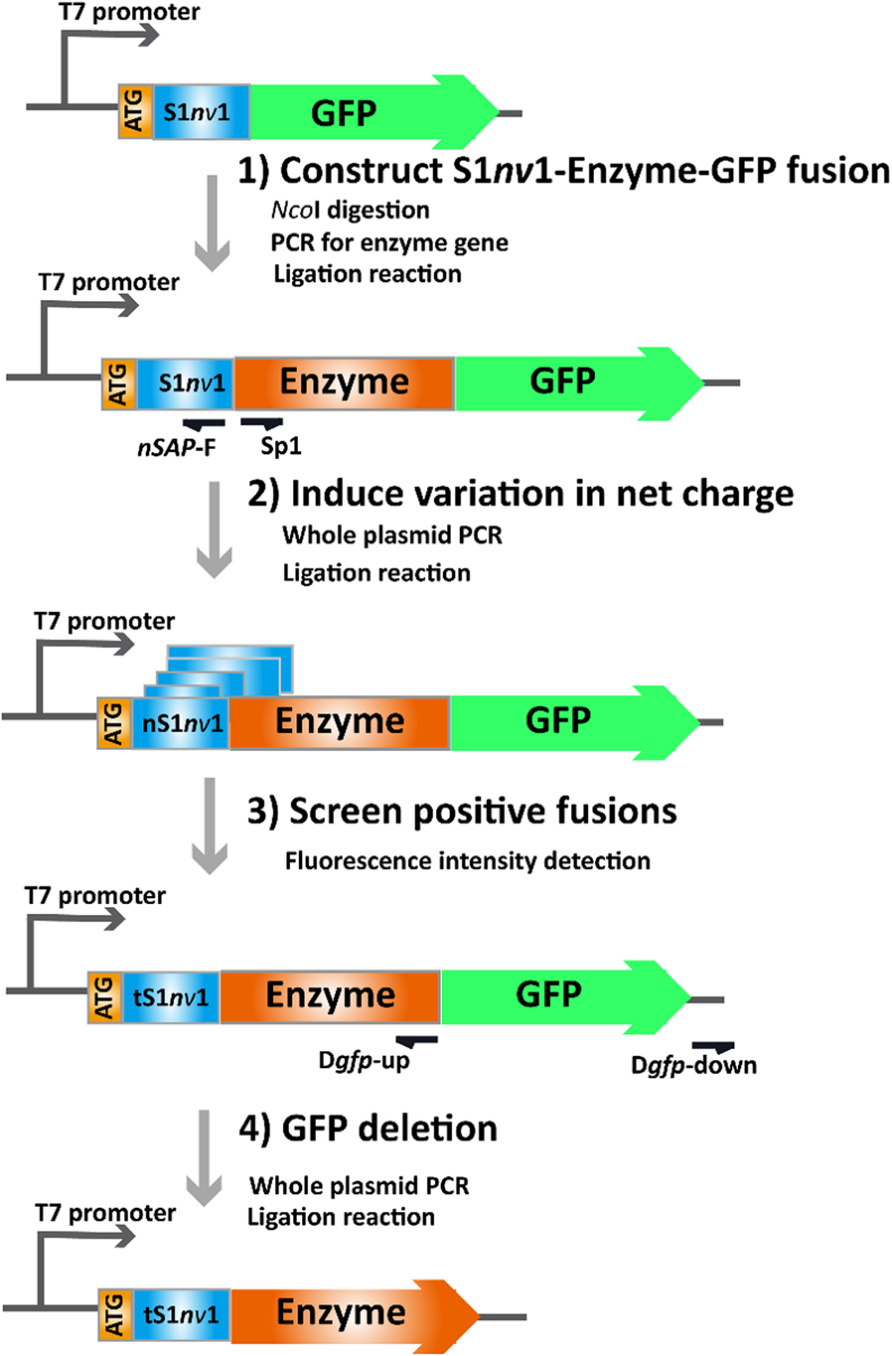
Schemes for construction of the expression tag library. The general scheme used to construct an expression tag library based on nS1*nv*1 (ANANARAR)_10_. The system and condition of PCR and ligation reaction, the screening method, and fluorescence intensity assays were performed and evaluated as described in “Materials and methods”

The target enzyme gene was cloned into the *Nco*I site of pET-22b(+)/*gfp*, yielding the plasmid expressing enzyme-GFP (wild-type enzyme fused with GFP) (Fig. 1d). Then the gene fragments of the enzyme were inserted into the *Nco*I site of the plasmid expressing GFP fused with S1*nv*1 (Fig. 1b), yielding the plasmid pET-22b(+)/*S1nv1*-*enzyme*-*gfp* expressing S1*nv*1-enzyme-GFP (enzyme fused with S1*nv*1 at the N-terminus and GFP at the C-terminus) (Fig. 2).

The general scheme for library construction is shown in Fig. 2. First, the charge mutation was introduced into the S1*nv*1 coding region of pET-22b(+) derivatives by a whole plasmid PCR, using a constant forward primer (*nSAP*-F) and the specific reverse primer *nSAP*-R. Second, the linearized fragments were ligated by T4 ligase (Shanghai, China), yielding the mixed plasmid libraries of pET-22b(+)/*nS1nv1*-*enzyme*-*gfp* expressing the nS1*nv*1-enzyme-GFP (enzyme fused with different units of S1*nv*1 at the N-terminus and GFP at the C-terminus). Third, a library of SAP fusions was constructed after protein production and detection based on RFU/OD_600_. Finally, after obtaining fusions with enhanced fluorescence intensity, the plasmids pET-22b(+)/*tS1nv1*-*enzyme*-*gfp* of the positive mutants were used as templates with specific forward primers D*gfp*-up and a constant reverse primer D*gfp*-down to remove the GFP gene, yielding the plasmids expressing recombinant enzymes fused with specific units of S1*nv*1. Specifically, the S1*nv*1 peptide for MTG was fused with a pro-peptide in its C-terminus [22].

### Culture conditions

The transformations containing the plasmids were transferred to Luria–Bertani (LB) media containing 100 μg/mL ampicillin. After overnight cultivation at 37 °C, 3% (v/v), *E. coli* seed cultures were inoculated into Terrific Broth (TB) medium containing 100 μg/mL ampicillin and cultivated at 37 °C. Upon reaching an optical density at 600 nm (OD_600_) of 0.6–0.8 in TB medium, protein production was induced by adding isopropyl β-d-1-thiogalactopyranoside (IPTG). Induction conditions in 96-well plates were as follows: PGL was induced for 4 h at 30 °C with 0.04 mM IPTG; LOX was induced for 8 h at 25 °C with 1 mM IPTG; ASN was induced for 8 h at 25 °C with 1 mM IPTG; and MTG was induced for 8 h at 25 °C with 0.05 mM IPTG. Induction conditions in shake flasks were as follows: PGL was induced for 24 h at 30 °C with 0.04 mM IPTG; LOX was induced for 24 h at 20 °C with 1 mM IPTG; ASN was induced for 12 h at 25 °C with 1 mM IPTG; and MTG was induced for 24 h at 25 °C with 0.05 mM IPTG.

### Fluorescent spectral analysis

The recombinant strains for GFP production or the fusion libraries cultured in the 96-wells or shake flasks under the corresponding culture conditions were harvested and washed twice by phosphate buffer solution (PBS, 50 mM, pH 7.5). Whole cell fluorescence and cell density (OD_600_) were measured on a Cytation 3 imaging reader system (BioTek, Winooski, VT, USA). The corresponding wild-type strain *E. coli* BL21 (DE3) was used as the negative control, and its fluorescence intensity was subtracted as the background. The emission and excitation wavelength of GFP were 520 and 488 nm, respectively.

### In Silico Analysis

The grand average of hydrophobicity (GRAVY, https://web.expasy.org/) was introduced to measure the hydrophobicity of SAPs. The increase in GRAVY value is an indication of strong hydrophobicity.

### Protein production and enzymatic properties determination

After shaker cultivation, the enzymatic activities of PGL, LOX, ASN, and MTG fusions were measured and purified as previously reported [23–26]. The production of the recombinant protein was determined from the specific and crude enzymatic activity of each protein.

The dynamic thermal stabilities of the pure enzymes were determined by measuring residual activity after incubating the enzyme solutions at the corresponding incubation temperature. The half-life (*t*_1⁄2_) was calculated using an exponential fitting of the data points [27]. The data were analyzed by fitting to first-order plots and the first-order rate constants (*k*_d_) were determined by linear regression of ln (residual activity) versus the incubation time (*t*). The time required for the residual activity to be reduced by half was calculated using the following equation: *t*_1/2_ = ln2/*k*_d_.

### Gel electrophoresis and protein concentration assay

The fermentation broth was centrifuged at 8000 rpm for 10 min, and the cell pellets were washed once by PBS (50 mM, pH 7.5) and re-suspended in 50 mM PBS (pH 7.5) containing 0.1 M NaCl. The re-suspended cell solution was prepared to have an absorbency of 8.0 at 600 nm. Then cells were lysed by ultra-sonication and the operational process was conducted as reported previously [17]. Aliquots of *E. coli* lysate, supernatant, and pellets (re-suspended fraction) were mixed with SDS loading buffer (NuPAGE1 LDS Sample Buffer 4×, Fisher Scientific) at a ratio of 3:1 and sodium dodecyl sulfate–polyacrylamide gel electrophoresis (SDS-PAGE) was performed as described previously [17].

## Results

### Effect of hydrophobic residues of SAP on GFP production

**Fig. 3:**
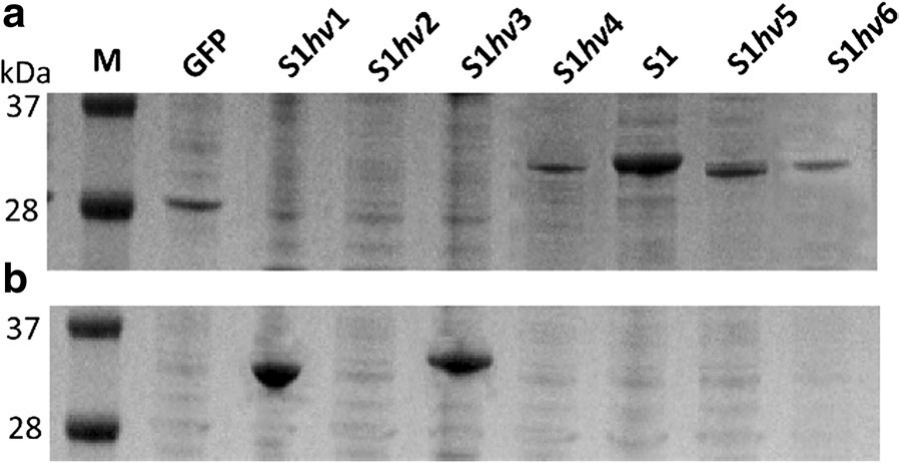
SDS-PAGE analysis of protein production by recombinant *E. coli*. **a** intracellular soluble fraction; **b** intracellular insoluble fraction; M: protein marker. The recombinant *E. coli* strains were grown in fermentation medium under corresponding culture conditions (see “Materials and methods”)

For expressing wild-type GFP from *A*. *Victoria* [20], the GFP gene was cloned into pET-22b(+) as shown in Fig. 1a. To consider the effects of SAP hydrophobicity on GFP production, the hydrophobic alanine (Ala) residues of S1 were replaced with isoleucine (Ile), leucine (Leu), valine (Val), phenylalanine (Phe), glycine (Gly), and proline (Pro) residues, yielding S1*hv*1, S1*hv*2, S1*hv*3, S1*hv*4, S1*hv*5, and S1*hv*6 (Table 1). The six S1 mutants showed GRAVY values ranges from 0.4 to − 2.65, suggesting a progressive variation in their hydrophobicity. Based on the constructs described in Fig. 1b, S1 and its mutants with different hydrophobicity were fused to the N-terminus of the GFP individually. Cells expressing the target proteins were harvested at the early exponential phase, and the GFP production presented as relative fluorescence intensity (fluorescence intensity divided by the corresponding OD_600_, RFU/OD_600_). The relative fluorescence intensity of each GFP fusion was divided by that of GFP without SAPs, yielding the normalized fluorescence intensity value. As shown in Table 1, in contrast to the wild-type GFP, the yield of the GFP fusions was enhanced by the SAPs with the hydrophobicity close to that of the S1 peptide. SDS-PAGE analysis indicated that two S1 variants with strong hydrophobicity (S1*hv*1 and S1*hv*3) induced the formation of insoluble inclusion bodies. The other fusions with S1 variants (S1*hv*2, S1*hv*4, S1*hv*5, and S1*hv*6) showed a remarkable decrease in soluble fraction in contrast to that of S1 (Fig. 3).

### Effect of hydrophilic residues of SAP on GFP production

**Fig. 4:**
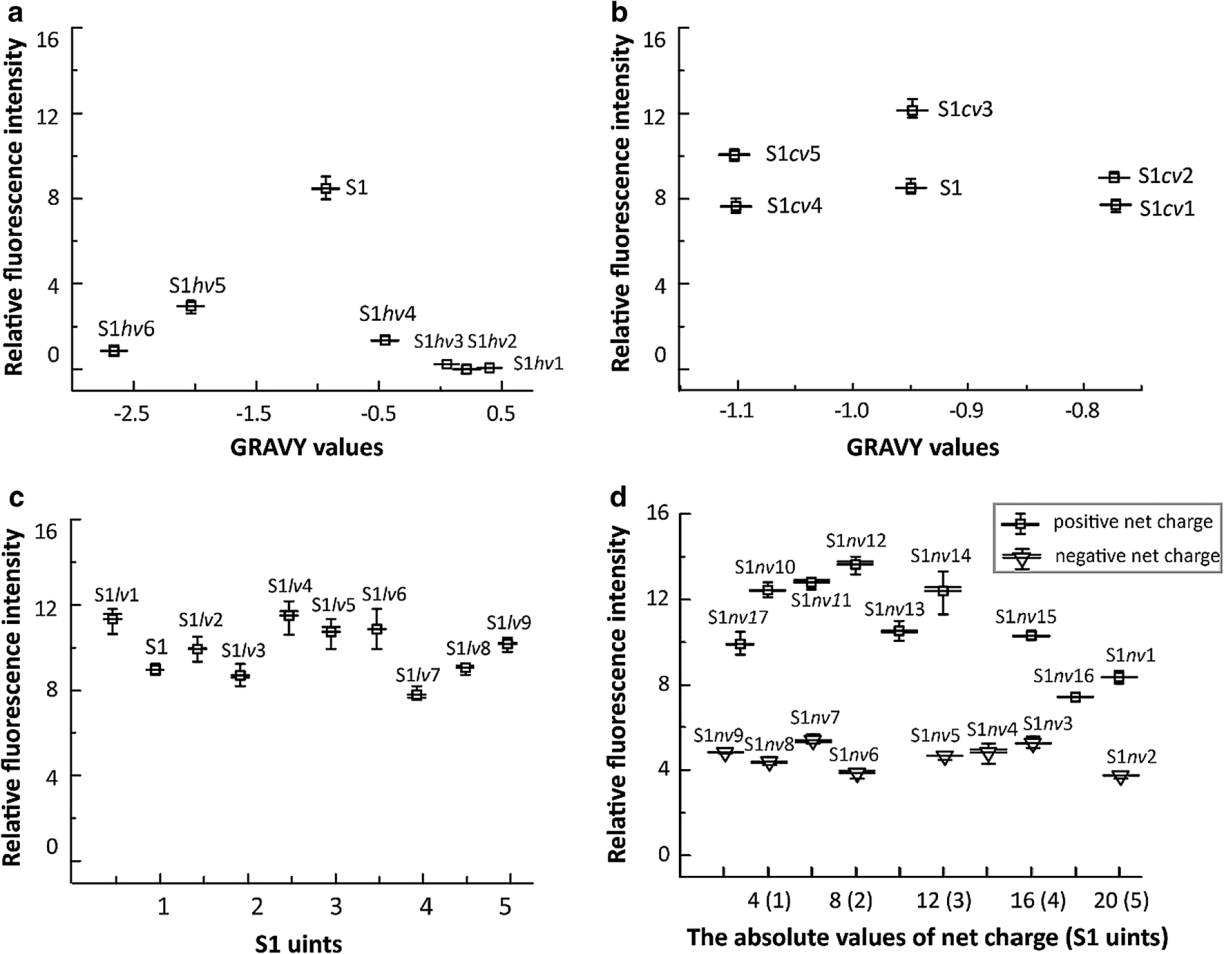
The fluorescence intensity of the GFP fusions with different SAPs. **a** GFP fused with S1 derivatives varied in hydrophobicity; **b** GFP fused with S1 derivatives varied in hydrophilic residues; **c** GFP fused with S1 derivatives varied in S1 units; **d** GFP fused with S1 derivatives varied in net charge. The parameters for each factor was summarized in Table 1. The fluorescence intensity of GFP fusions was normalized by that of GFP. Each result was the average value of three parallel experiments

To understand the role of hydrophilic residues in SAPs, S1 variants with different hydrophilic residues were synthesized, including S1*cv*1 (glutamic acid and lysine residues were replaced by aspartic acid and histidine residues, respectively), S1*cv*2 (lysine residues were replaced by histidine residues), S1*cv*3 (glutamic acid residues were replaced by aspartic acid residues), S1*cv*4 (lysine residues were replaced by arginine residues), and S1*cv*5 (glutamic acid and lysine residues were replaced by aspartic acid and arginine residues, respectively) (Table 1). These SAPs were then fused separately to the N-terminus of the GFP (Fig. 1b). As described in Fig. 4b, all the SAP fusions showed remarkable increases in fluorescence intensity compared with that of GFP, and the S1 variants with the same hydrophobicity displayed different fluorescence intensity. In contrast to S1 variants with changed hydrophilic residues (Fig. 4a), the fluorescence intensity of each fusion here exhibited relative lower fluctuation. These results suggested that the type of hydrophilic residues have little effect on the efficiency of the SAPs.

### Effect of SAP length on GFP production

To investigate how the length of SAPs influences protein production, the S1 variants with 5, 1/2, 3/2, 2, 5/2, 3, 7/2, 4, and 9/2 S1 units were generated through PCR (Additional file 1: Fig. S1), and named S1*lv*1, S1*lv*2, S1*lv*3, S1*lv*4, S1*lv*5, S1*lv*6, S1*lv*7, S1*lv*8, and S1*lv*9, respectively. As shown in Fig. 1b, each S1 variant was fused to the N-terminus of GFP. It was found that the fluorescence intensities of GFP fusions were not changed greatly by the extension of S1 units, indicating that SAP length only had a slight effect on protein production (Fig. 4).

### Effect of the SAP net charge on GFP production

To achieve a variation in the net charge of SAPs, a PCR procedure (Fig. S1) was conducted using the SAP units (ANANARAR)_10_ and (ANANADAD)_10_ as templates, yielding SAPs carrying net charge values ranging from + 4 to + 20 and from − 2 to − 20 (Table 1). For unknown reasons, we could not obtain SAPs with net charges of − 18, − 10, + 2, and + 14 using the PCR procedure (Table 1). Each SAP with a varied net charge was fused to the N-terminus of GFP (Fig. 1b). As shown in Fig. 4, all of the SAPs with different net charges increased GFP production. Under the same SAP length, SAPs with positive net charges produced the fluorescence intensities of GFP fusions 0.95–2.52-times higher than those of SAPs with negative net charges. In particular, the fluorescence intensities of the GFP fused with those SAPs carrying net charges ranging from + 4 to + 16 were over tenfold higher than that of GFP. The SAP Hence, regulating the net charge of the SAPs could effectively improve the production of SAP fusions.

### Effect of linker on GFP production

Previous studies [28] have reported that the length and flexibility of linker peptides exert an important influence on the structure or production of the fusion proteins. Generally, a combination of the flexible (GGGGS) and rigid (EAAAK) linker units could generate linker peptides which vary in length and flexibility [29]. To examine the effect of linker length on GFP fusion production, S1 was fused to the N-terminus of the GFP via the linker peptides composed of 1 to 5 units of rigid or flexible linker units, respectively (Table 2, Fig. 1c). It was shown that the production of the GFP fusions was not changed greatly with variation in the length of flexible and rigid linker peptides (Additional file 1: Fig. S2A). To investigate the flexibility effects, the GFP was fused with S1 via linker peptides that were totally composed of 5 linker units and varied in the ratio of flexible (GGGGS) and rigid (EAAAK) units (Table 2, Fig. 1c). Similarly, changes in flexibility have little effect on the production of GFP fusions (Additional file 1: Fig. S2B).

### SAP library construction for enhanced protein production

Based on key factor analysis, the variables of the SAP library for protein expression were downsized to the number of net charges. Because SAPs with positive net charges were more effective than those with negative net charges, when related to protein expression, the net charges of the SAPs were restricted to the values ranging from + 1 to + 20.

The gene of the target enzyme was inserted into the gene of S1*nv*1-GFP, resulting in plasmids expressing S1*nv*1-enzyme-GFP, then fusions with SAP carrying different positive charges were generated by a PCR procedure using plasmids expressing S1*nv*1-enzyme-GFP as a template. The scheme for constructing the library is illustrated in Fig. 2.

### Evaluation of the SAP library using four enzymes

**Fig. 5:**
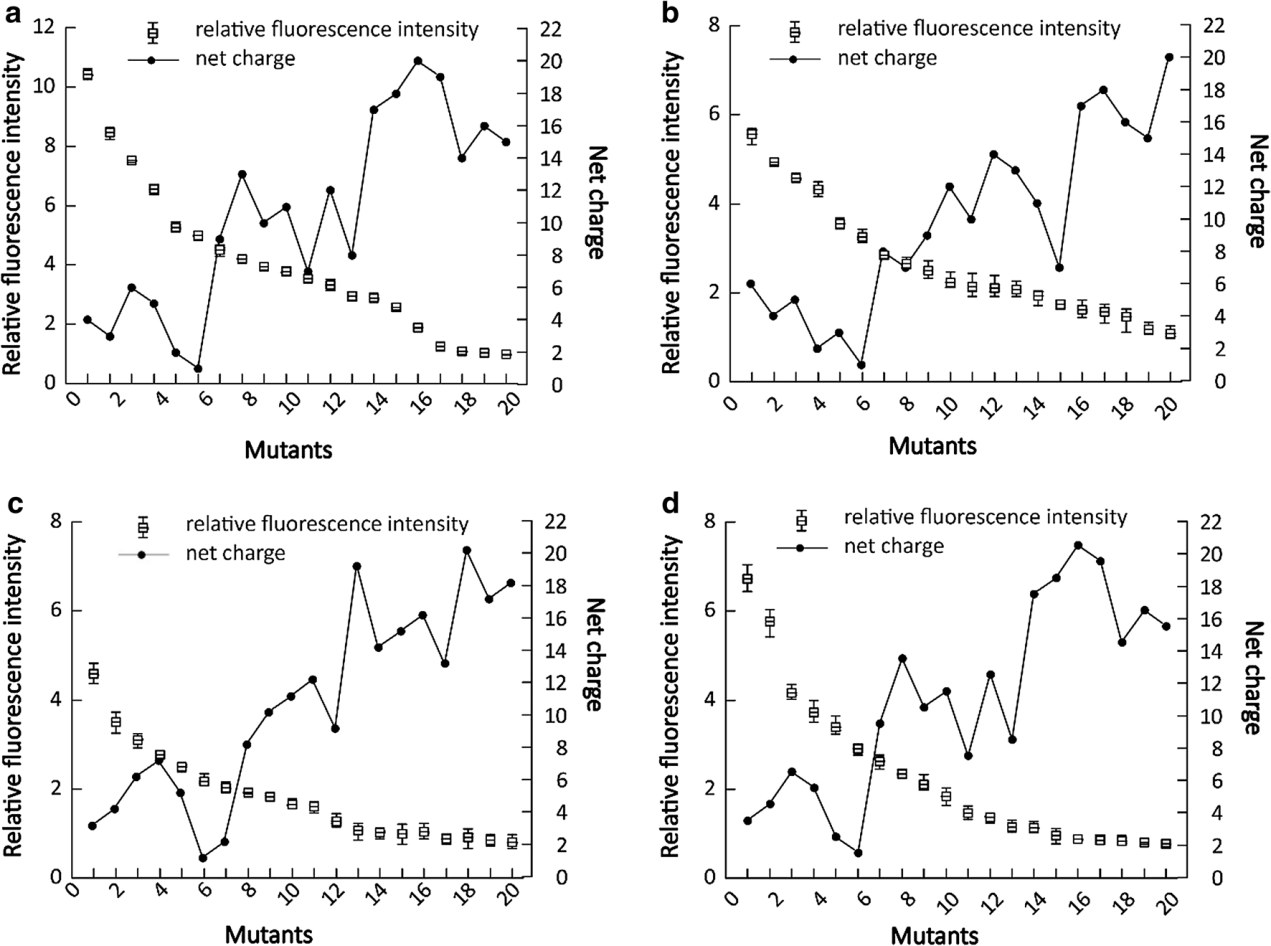
Fluorescence intensity and SAP net charge of each enzyme fused with SAP and GFP. **a** PGL fusions; **b** LOX fusions; **c** ASN fusions; **d** MTG fusions. The relative fluorescence intensity of each fusion was divided by that of the corresponding wild-type enzyme, yielding the normalized relative production yield. Each result was the average value of three parallel experiments

**Fig. 6:**
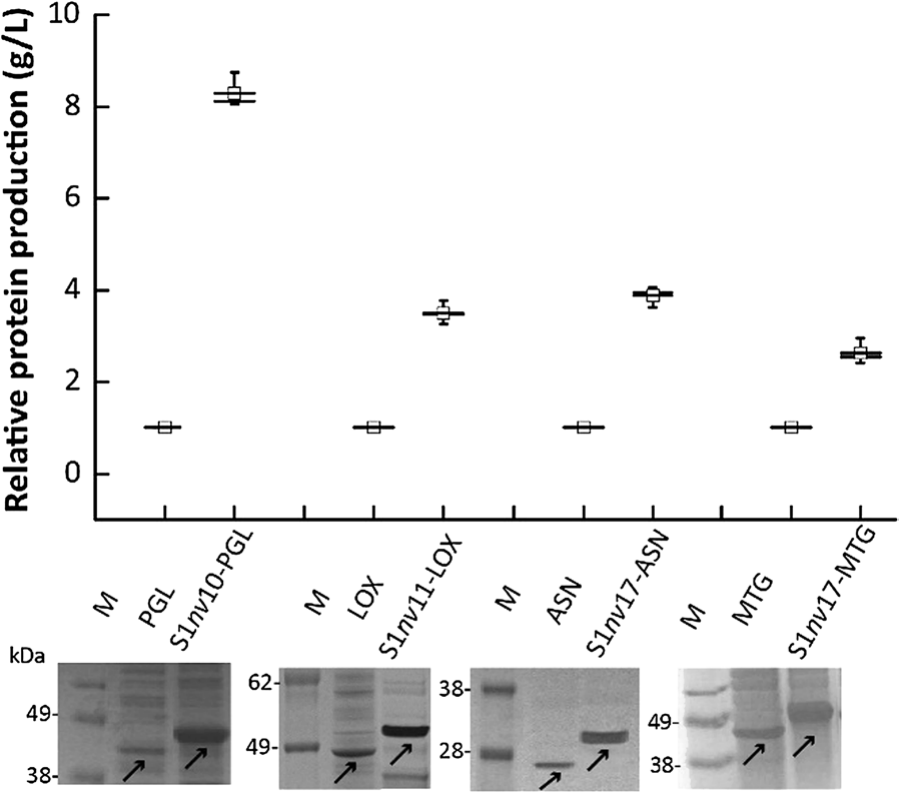
Analysis of the protein production of enzymes solely fused with SAP at N-terminus in recombinant *E. coli*. S1*nv*10-PGL: the PGL fused with S1*nv*10; S1*nv*11-LOX: LOX fused with S1*nv*11; S1*nv*17-ASN: ASN fused with S1*nv*17; S1*nv*17-MTG: MTG fused with S1*nv*17. The relative protein production of each fusion was divided by that of the corresponding wild-type, yielding the normalized relative production yield. In SDS-PAGE analysis M represents the protein marker and the arrow indicates the target band. Each result was the average value of three parallel experiments

Four enzymes, which included PGL from *B*. *Subtilis* WSHB04-02 [30], LOX from *P. Aeruginosa* BBE [23], MTG from *S*. *Mobaraense* [25], and ASN from *E. coli* strain BL21 (DE3) [31], were used to verify the efficiency of the library. Each enzyme fused with GFP was constructed (Fig. 1d) and used as the control sample*.* As shown in Fig. 5, all of the cells expressing enzymes fused with SAP and GFP exhibited enhanced fluorescence intensity in contrast to cells carrying the corresponding enzyme fused with GFP. Moreover, the SAPs that were screened covered the net charges ranging from + 1 to + 20, and those SAPs with net charge + 4 (S1*nv*10), + 6 (S1*nv*11), + 3 (S1*nv*17), and + 3 (S1*nv*17) (Table 1) achieved the highest fluorescence intensity with PGL, LOX, ASN, and MTG, respectively. To confirm the positive relationship between the fluorescence intensity and protein production, the GFP tag was removed from fusions with SAP and GFP for each enzyme, yielding the enzyme solely fused with SAP (Fig. 2). As shown in Additional file 1: Figure S3, the yield of enzymes fused with SAPs displayed a substantial positive correlation with fluorescence intensity of the corresponding enzyme fusion with SAP and GFP in the case of the four enzymes (PGL, LOX, ASN, and MTG). Moreover, as shown in Fig. 6, in contrast to the corresponding wild-type enzyme, the PGL fused with S1*nv*10, LOX fused with S1*nv*11, ASN fused with S1*nv*17, and MTG fused with S1*nv*17 exhibited 8.3, 3.5, 3.68, and 2.64-fold increases in protein yield, respectively. SDS-PAGE analysis showed the same trend in the protein expression of wild-type and SAP-fusion of each enzyme (Fig. 6).

### Enzyme characterization of the SAP fusions

The influence of SAPs on the catalytic efficiencies and thermal stabilities of the fusions were also assayed. As shown in Table 3, the specific activities changed slightly whereas the thermal stabilities of the SAP fusions improved significantly. The specific activities of S1*nv*10-PGL, S1*nv*11-LOX, and S1*nv*17-ASN presented 0.79, 0.26, and 0.23-fold increase in specific activity, and 2.1, 3.82, and 0.98-fold increase in *t*_1⁄2_ relative to the corresponding wild-type, respectively. For MTG, because the active enzyme was produced by the removal of its N-terminal pro-peptide, the enzymatic properties of the fusions were nearly not changed compared with the wild-type (data were not shown).

**Table 3:** Enzymatic properties of wild-type enzymes and fusion proteins

Enzyme	Value ± SD			
	Km (g L^−1^#, mM*, μmol L^−1^″)	kcat (min^−1^#, S^−1^*, S^−1^″)	Specific activity (U mg^−1^)	t1/2 (min)
PGL	0.27 ± 0.011	12.69 ± 0.4	279.14 ± 3.5	5.3 ± 0.32
S1*nv*10-PGL	0.47 ± 0.07	65.6 ± 6.8	499.6 ± 8.4	16.4 ± 1.3
LOX	0.057 ± 0.004	24.7 ± 2.97	30.2 ± 1.22	9.7 ± 0.43
S1*nv*11-LOX	0.052 ± 0.006	81.2 ± 3.6	38.23 ± 1.87	46.8 ± 1.5
ASN	14.2 ± 0.31	49.2 ± 1.9	17.34 ± 0.16	12.3 ± 0.42
S1*nv*17-ASN	21.4 ± 0.34	68.6 ± 2.5	21.34 ± 0.6	24.4 ± 0.65

Each result was the average value of three parallel experiments. SD is the standard deviation of three experiments

^#^, * and " indicated the units for PGL, LOX, and ASN, respectively

## Discussion

Traditional optimization strategies, based on promoters, ribosome-binding sites, or untranslated region modifications, have been used to improve the production of recombinant enzymes [32, 33]. It should be noted, that some proteins remain poorly expressed under these strategies probably due to differences in amino acid sequence [34]. To solve this problem, sequence modification [10] of the target protein has received much attention in recent years, including the addition of expression fusion tags to the N-terminus of target proteins [35]. However, the fusion of proteins with expression tags could not improve the production of all proteins [36]. SAPs, an emerging fusion tag, have been successfully used for improving protein production [17], purification [37], and thermal stability or catalytic activity [14]. In this study, an expression tag library composed of SAPs, which varied in net charge, was constructed in *E. coli*. The usefulness of this library was validated by expressing PGL, LOX, ASN, and MTG, with their optimized SAP fusions exhibiting 8.3, 3.5, 2.64, and 3.68-fold increases in production yield, respectively, relative to the corresponding wild-type enzyme. Thus, the results indicated the high efficiency of this SAP-based library in *E. coli*.

Key factor analysis indicated that hydrophobicity and net charges of SAPs played a key role in SAP fusion expression. It was shown that changes in the hydrophobicity of S1 tend to cause the formation of insoluble inclusion bodies of protein. Moreover, the SAP positive net charge was more efficient for protein expression than those with a negative net charge. Thus, the SAP tag library only included 20 types of SAPs with net charges ranging from + 1 to + 20. Because the SAP length had little effect on the expression of SAP fusions, the variation in SAP net charge was achieved by altering the length of the positive charged S1*nv*1 (ANANARAR)_10_ through a PCR procedure, thereby, avoiding the tedious gene cloning of each SAP fusion one by one. Although only 96 colonies of each enzyme fusion were screened, the SAP library still achieved a considerable rate of those fusions with enhanced protein expression, which was much higher than that of irrational-screening strategies [38]. Moreover, in contrast to N-terminal modification [39] or site-directed mutagenesis strategies [40], application of the SAP library could also improve the thermal stabilities or activities of the enzymes. Due to the application of single-factor experiment, the synergistic effect among different factors were not considered in the current study, such as the effect of the SAP hydrophobicity on the protein expression under the optimized net charges in SAPs. Further investigation will be performed on the effects of different combinations of the four factors.

In this study, the S1 variants with a hydrophobicity that was lower or higher than S1 showed reduced fusion protein expression. To date, the mechanism for the effect of hydrophobicity on protein expression is still unclear. Among the six S1 hydrophobicity variants, SAPs with Ile and Leu induced the GFP inclusion body formation. This result was consistent with a previous study in which active inclusion bodies were obtained after fusions with a SAP containing several Leu residues [41]. Thus, the formation of inclusion bodies may partly account for reduced expression. Considering the high sensitivity of the fusion expression to SAP hydrophobicity, further investigation will be performed on the precise regulation of SAP hydrophobicity to improve the diversity of the SAP library.

As indicated by the key factor analysis, the positive net charge of SAPs could enhance the protein production more effectively in contrast to those with no or negative net charges. It has been reported that the positive charge on the N-terminus of newly synthesized peptides could interact with the negatively charged ribosomal exit tunnel to slow down the initiation rate of protein expression [42]. The relatively low initiation rate may benefit correct protein folding through facilitating interactions between the emerging peptide and the chaperone proteins, and these interactions may increase with the number of positively charged residues [43]. As SAP was fused at the N-terminus of the target proteins, it was possible that SAPs with positive net charges enhanced the protein expression through similar electronic interactions. However, the optimal number of the net charge in SAPs for protein expression ranged from + 2 to + 6 among the four enzymes tested, and the additive effects on the expression were not observed. Notably, the N-terminal sequence with the ability to affect the protein expression was approximately limited to the first 18 amino acid residues (around) [42]. Because the variation in positive net charge was obtained by changing the length of the positive charged S1*nv*1 (ANANARAR)_10_, the S1*nv*1 variants with over six positive net charges were composed of more than 24 residues. The positively charged residues after the first 18 amino acid residues of the SAP may reduce the expression efficiency due to the additional interactions with the charged ribosomal exit tunnel. Increasing positively charged residues without extending the SAP sequence may further enhance fusion expression.

## Conclusion

In summary, we proposed an efficient expression tag library based on SAPs in *E. coli*, where a positive net charge was selected as the major variable. As a proof-of-concept, four enzymes showed substantial increases in protein production without sacrificing specific activities and thermal stabilities, suggesting that the strategy was efficient. This study provided a simple and convenient strategy to enhance the production of recombinant proteins and established a basis for the application of SAP fusion.

## Additional file

**Additional file 1: Table S1.** Primers used in this study. **Figure S1.** Schemes for the construction of GFP fused with SAP varied in S1 unit. In the first round of PCR, the forward primers S1*lv*1-F separately bind to four sites of the S1 encoding region (1, 2, 3, and 4) under low annealing temperature, yielding I, II, III, and IV linearized plasmids. Then in the second round of PCR, these linearized plasmids are used as the templates to amplify the linearized plasmids encoding GFP fused with different lengths of SAPs. After the ligation reaction, the circular plasmids encoding GFP fused with nS1 are obtained. The PCR and ligation reaction system and conditions are performed as described in Materials and Methods. **Figure S2.** The fluorescence intensity of GFP fused with S1 via different linker composition in *E. coli*. (A) GFP fused with S1 via different linker units; (B) GFP fused with S1 via 5 linker units containing a different number of flexible linker units. All linker sequences are listed in Table 2. The rigid and flexible linker units referred to EAAAK and GGGGS, respectively. The fluorescence intensity of GFP fusions was normalized by that of GFP. Each result was the average value of three parallel experiments. **Figure S3.** The correlation analysis of protein production and fluorescence intensity. (A): PGL and its GFP fusions; (B): LOX and its GFP fusions; (C): ASN and its GFP fusions; (D): MTG and its GFP fusions. The relative protein production and relative fluorescence intensity were measured as described in Materials and Methods. Each result was the average value of three parallel experiments.

## Data Availability

All data generated or analyzed during this study are included in this published article and additional file.

## Abbreviations

SAPs: self-assembling amphipathic peptides
PGL: poly galacturonate lyase
LOX: lipoxygenase
ASN: l-asparaginase
MTG: transglutaminase
*E. coli*: *Escherichia coli*
