# Supplementary material for: An efficient expression tag library based on self-assembling amphipathic peptides
Source: Microb Cell Fact. 2019 May 27;18:91. doi: 10.1186/s12934-019-1142-9 (PMC6535861; doi:10.1186/s12934-019-1142-9)
Supplement: Supplementary file 1 — Additional file 1: Table S1. Primers used in this study. Figure S1. Schemes for the construction of GFP fused with SAP varied in S1 unit. In the first round of PCR, the forward primers S1lv1-F separately bind to four sites of the S1 encoding region (1, 2, 3, and 4) under low annealing temperature, yielding I, II, III, and IV linearized plasmids. Then in the second round of PCR, these linearized plasmids are used as the templates to amplify the linearized plasmids encoding GFP fused with different lengths of SAPs. After the ligation reaction, the circular plasmids encoding GFP fused with nS1 are obtained. The PCR and ligation reaction system and conditions are performed as described in Materials and Methods. Figure S2. The fluorescence intensity of GFP fused with S1 via different linker composition in E. coli. (A) GFP fused with S1 via different linker units; (B) GFP fused with S1 via 5 linker units containing a different number of flexible linker units. All linker sequences are listed in Table 2. The rigid and flexible linker units referred to EAAAK and GGGGS, respectively. The fluorescence intensity of GFP fusions was normalized by that of GFP. Each result was the average value of three parallel experiments. Figure S3. The correlation analysis of protein production and fluorescence intensity. (A): PGL and its GFP fusions; (B): LOX and its GFP fusions; (C): ASN and its GFP fusions; (D): MTG and its GFP fusions. The relative protein production and relative fluorescence intensity were measured as described in Materials and Methods. Each result was the average value of three parallel experiments. [file 12934_2019_1142_MOESM1_ESM.docx]

**Table S1.** Primers used in this study.

| Primers | Sequence (5’-3’) | Notes |
| --- | --- | --- |
| S1*lv*1-F | TTYAGCTTYAGCTTYCGCTTYCGCTTYGGCTTYCGCTTYTGCTTYTGC |  |
| S1*nv*1-F | AYKAGCAYKAGCAYKCGCAYKCGCAYKGGCAYKCGCAYKTGCAYKTGC |  |
| S1*nv*2-F | GCARATGCARATGCGRATGCCRATGCGRATGCGRATGCTRATGCTRAT |  |
| *S1*-R | ATGGGTAAGGGAGAAGAACTTTTC | constant |
| *pgl*-F | CGCCATATGATGGATGCTGATTTAGGCCAC |  |
| *pgl*-R | CCGCTCGAGTTAATTTAATTTACCCGCAC |  |
| *lox*-F | CGCCATATGAATGACTCGATATTCT |  |
| *lox*-R | CCGCTCGAGTGCGGCCGCAAGCTTTCAG |  |
| *asn*-F | CGCCATATGGAGTTTTTCAAAAAGACG |  |
| *asn*-R | CCGCTCGAGTTAGTACTGATTGAAGATCTGCTG |  |
| *mtg*-F | GCCATGGATGACAATGGCGCGGGGG |  |
| *mtg*-R | GTGCTCGAGCGGCCAGCCCTGCTTTA |  |
| *gfp*-F1 | ATGGGTAAGGGAGAAGAAC |  |
| *gfp*-R1 | TTATTTGTATAGTTCATCCATGCCATG |  |
| *nSAP*-F | AYKAGCAYKAGCAYKCGCAYKCGC | constant |
| *nSAP*-R*PGL* | GCCATGGATGCTGATTTAGGCCACCAGACGTTGGGA | specific for PGL |
| *nSAP*-R*LOX* | GCTAAGGCTAAAGAATTCGCCATGGATAATGACTCG | specific for LOX |
| *nSAP*-R*ASN* | CATGCCATGGAGTTTTTCAAAAAGACGGCACTT | specific for ASN |
| *nSAP*-R*MTG* | ATGGATGACAATGGCGCGGGGGAAGAGACGAAGTC | specific for MTG |
| D*gfp*-*pgl*-up | ATTTAATTTACCCGCACCCGCTTGATTTATGACATT | specific for PGL |
| D*gfp*-*lox*-up | GATATTGGTGCTCGCCGGGATACGGCTCGGCAACAG | specific for LOX |
| D*gfp*-*asn*-up | GTACTGATTGAAGATTTGCTGGATCTGCTGCGGATC | specific for ASN |
| D*gfp*-*mtg*-up | TCACTCGAGCGGCCAGCCCTGCTTTACCTTGTCG | specific for MTG |
| D*gfp*-down | TAATCGAGCTTAAGGGAGTCG | constant |


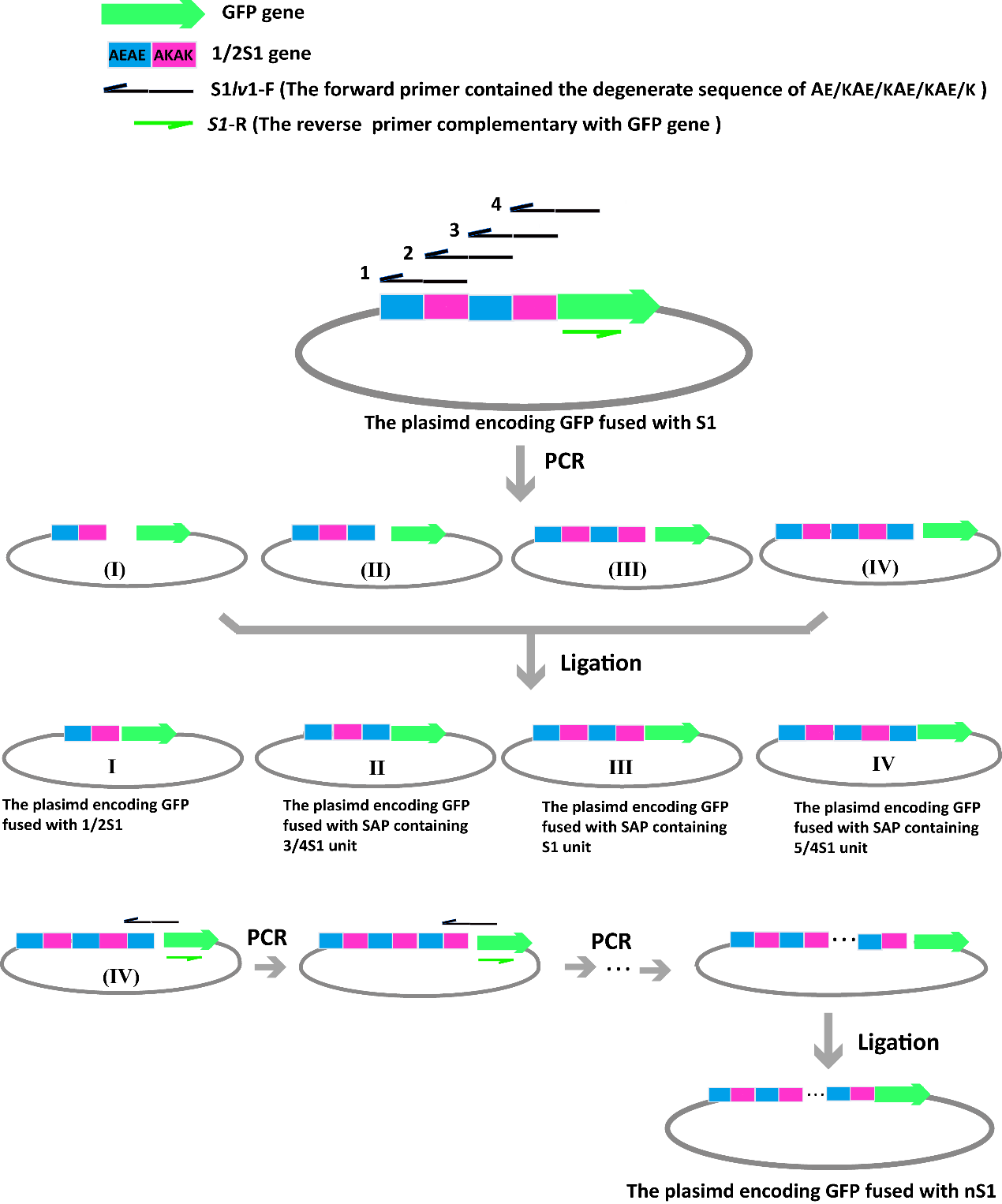


**Figure S1.** Schemes for the construction of GFP fused with SAP varied in S1 unit. In the first round of PCR, the forward primers S1*lv*1-F separately bind to four sites of the S1 encoding region (1, 2, 3, and 4) under low annealing temperature, yielding I, II, III, and IV linearized plasmids. Then in the second round of PCR, these linearized plasmids are used as the templates to amplify the linearized plasmids encoding GFP fused with different lengths of SAPs. After the ligation reaction, the circular plasmids encoding GFP fused with nS1 are obtained. The PCR and ligation reaction system and conditions are performed as described in the Materials and Methods.


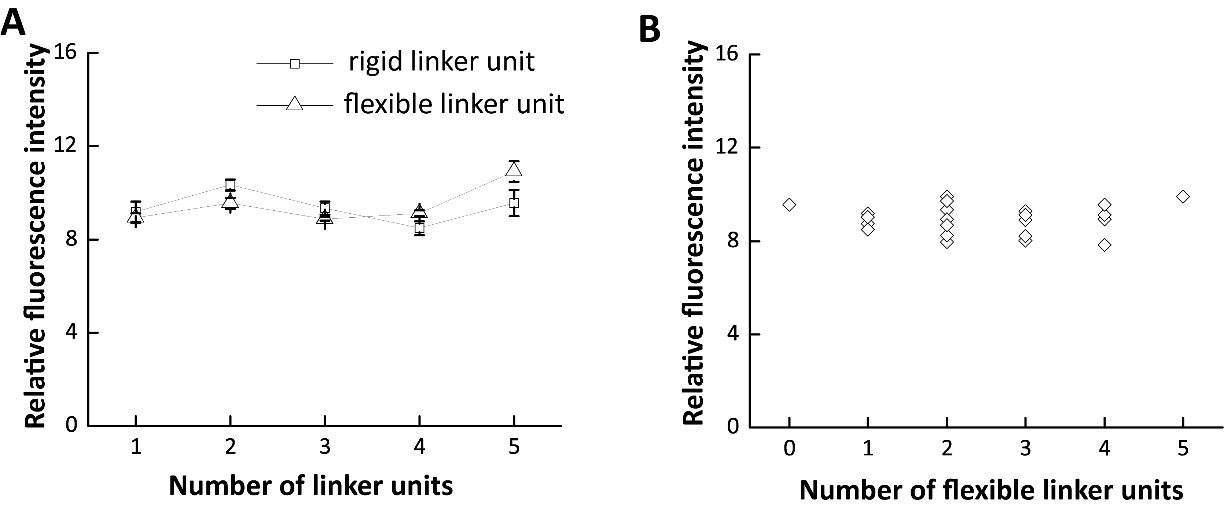


**Figure S2.** The fluorescence intensity of GFP fused with S1 via different linker composition in *E. coli*. (A) GFP fused with S1 via different linker units; (B) GFP fused with S1 via 5 linker units containing a different number of flexible linker units. All linker sequences are listed in Table 2. The rigid and flexible linker units referred to EAAAK and GGGGS, respectively. The fluorescence intensity of GFP fusions was normalized by that of GFP. Each result was the average value of three parallel experiments.


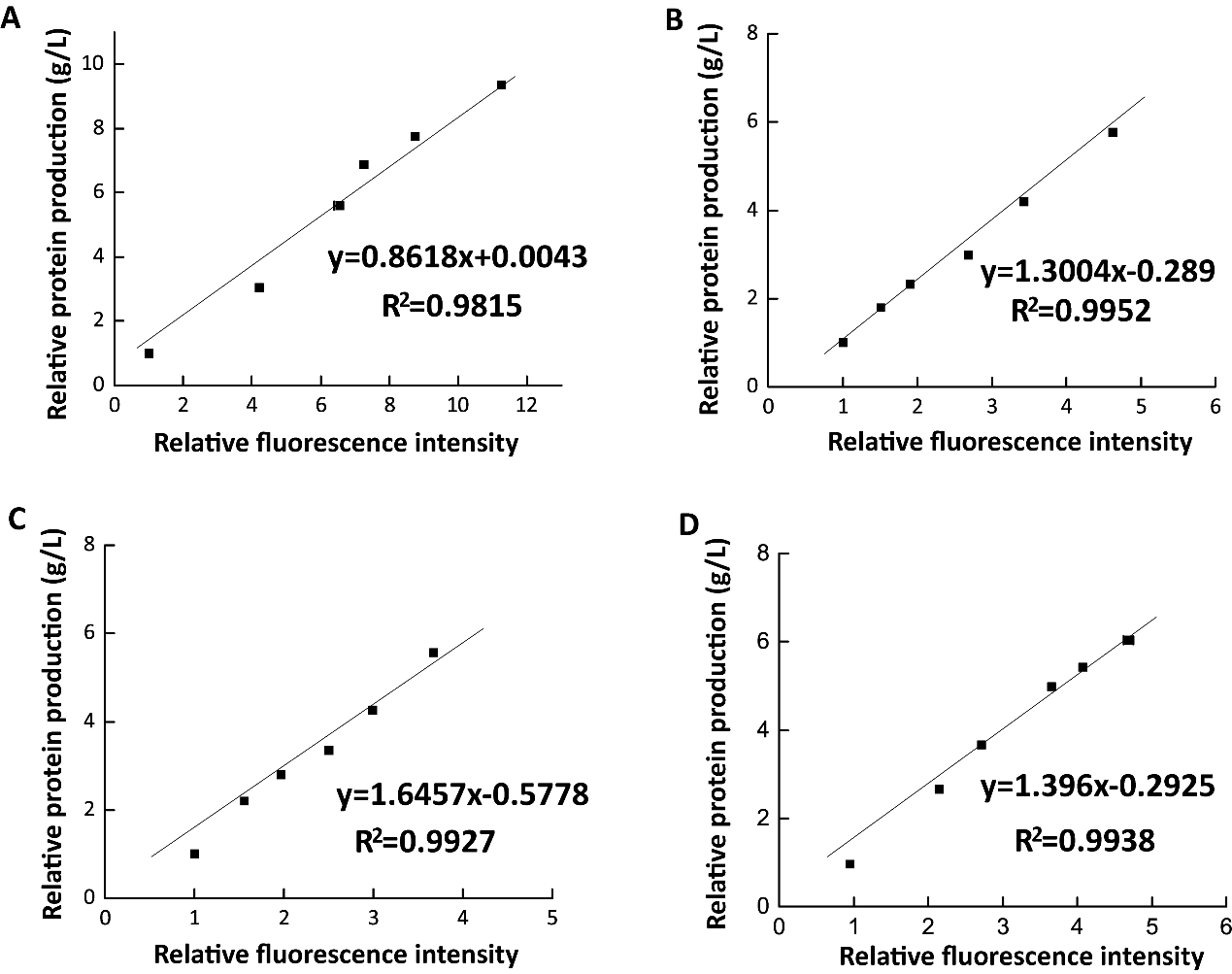


**Figure S3.** The correlation analysis of protein production and fluorescence intensity. (A): PGL and its GFP fusions; (B): LOX and its GFP fusions; (C): ASN and its GFP fusions; (D): MTG and its GFP fusions. The relative protein production and relative fluorescence intensity were measured as described in Materials and Methods. Each result was the average value of three parallel experiments.
